# Histopathological Evaluation of Behçet's Disease and Identification of New Skin Lesions

**DOI:** 10.1155/2012/209316

**Published:** 2011-10-18

**Authors:** Özgür Gündüz

**Affiliations:** Department of Dermatology, Faculty of Medicine, Kırıkkale University, 71100 Kırıkkale, Turkey

## Abstract

Behçet's disease (BD) is a multisystemic, relapsing inflammatory disorder with an obscure etiology and pathogenesis. Diagnosis depends on the clinician's ability to identify a group of nonspecific mucocutaneous lesions, which also manifest in a number of other diseases. In recent years, there has been an increase in the studies focusing on the histopathological aspects of Behçet's disease diagnostic mucocutaneous lesions. Their results emphasize the value of histopathology and direct immunofluorescence (DIF) in the differential diagnosis of Behçet's disease.

## 1. Introduction

After seventy-four years, since Dr. Hulusi Behçet had published his classic paper describing the three major signs [1], BD are still an enigma for clinicians and researchers. Almost all aspects of BD is a source of debate and even its diagnostic criteria, classification, and pathogenesis are controversial [2–4]. 

In the last two decades, extensive studies have been conducted to reveal the nature of BD. In the light of these studies, BD is now recognized as a chronic, multisystemic vasculitis [2, 5–7]. Whether this vasculitis is a result of autoimmunity is controversial [2, 3], but there is increasing evidence indicating the possible role of immunologic mechanisms in the pathogenesis. Evaluation of lesion-free skin and mucocutaneous lesions of patients with BD patients by DIF reveals immunoreactant deposits on the vessel walls [8–11]. Also, elevated serum levels of several proinflammatory cytokines (IL-1, IL-4, IL-6, TNF-*α*, etc.) have been reported in these patients [12]. A special subgroup of T lymphocytes (*γδ* T lymphocytes), which play an important role in mucosal immunity, are found to be present in the increased numbers in circulation and mucosal lesions of patients [13]. Several research groups found out that cultured *γδ* T lymphocyte cells proliferate when stimulated with mycobacterial heat shock proteins and products of several oral pathogen microorganisms [13, 14]. 

Correlation between the hyperactive state of neutrophils and BD activity is another well-known fact. The underlying mechanism is unknown. Antigen-presenting cell and T-lymphocyte-derived cytokines and chemokines are believed to be responsible for neutrophiles hyperreactivitiy [15].

BD is mostly encountered in the Mediterranean and Middle and Far East countries, Turkey having the highest prevalence with 80–420 cases/100.000 [3, 16]. 

Genetic studies show statistically significant association with HLA-B51 [17, 18].

Antiendothelial antigens are another immunological anomaly found in BD patients, but there is no solid evidence for their participation in the pathogenesis [19].

Considering available information, it is no surprise that most of the working hypotheses for the BD pathogenesis point out an external factor (an infectious or regional pathogen, i.e., Parvovirus B19 [20], Helicobacter pylori [21], *Streptococcus sanguis* [22], etc.). This factor probably stimulates an abnormal immune reaction, during which certain types of lymphocytes are stimulated and neutrophils reach a hyperreactive state in the people with a genetic predisposition. Diagnosis of BD depends on the presence of several clinical findings. These findings are defined by the International Study Group (ISG) of Behçet's Disease and published in 1990 [23] (Table 1). Major weak points of ISG criteria are the occasional long intervals between manifestations of mucocutaneous lesions with the diagnostic value and lack of their specificity. Similar lesions may manifest in an extensive number of diseases (e.g., papulopustular lesions, erythema nodosum). Knowledge of histopathological features of BD mucocutaneous lesions may be beneficial for differential and early diagnosis. 

## 2. Aphthous Oral Ulcers

Aphtous oral mucosa ulcers (AOUs) are usually the initial clinical manifestation [4, 24]. Ideguchi et al. evaluated 412 BD patients' hospital records covering 16 years follow-up data [4]. The result of this study revealed that in some cases AOU had proceeded 10 years a definitive diagnosis. Unfortunately, AOUs are neither specific nor rare. Similar ulcers may be observed during the course of several systemic or local diseases, including inflammatory bowel diseases (Crohn), Sweet syndrome, cyclic neutropenia, and herpes infections. If recognized by clinican, other clinicial manifestations of this disease may enable a quick differential diagnosis. 

Regardless of the concurrent disease, morphologies of AOU are similar. An AOU has sharp borders surrounded by an erythematous rim and a base covered with yellow-white coloured pseudomembrane. AOUs are classified into three groups according to ulcer diameter, but these three morphological forms are recognized as parts of the same spectrum.

1. Minor aphthae are shallow mucosal ulcers with a diameter <10 mm, which may be found in groups, usually on nonkeratinized section of oral mucosa (lateral sides and ventral surface of tongue, mouth floor).
2. Major aphtous ulcers have similar morphologies, but they have larger diameter (>10 mm), are deeper than the minor variants, and tend to heal with scarring.
3. Herpetiform aphthae are pinpoint shaped, very small and shallow mucosal ulcers and tend to occur in crops. Sometimes they may converge and form large ulcers with irregular borders.

Several pathogenetic mechanisms for aphtous ulcers have been proposed such as T-cell-mediated immunologic reactions, inhibition of mucosal healing by cytokines, nutritional (vitamin B_12_, folic acid) deficiencies, and viral or bacterial assault, but neither of these are proven. 

 To include oral aphtous ulcers in the diagnostic criteria of BD, at minimum three episodes should be observed in one year. Since a definitive BD diagnosis requires the other mucocutaneous features to develop, patients may undergo a long prediagnostic interval, manifesting only oral aphthae. Recurrent aphtous stomatitis (RAS), the most common type of oral aphthae [25–28], affects nearly one quarter of the world population [1] and runs a similar course to the prediagnostic phase of BD, providing a diagnostic challenge for the clinician. RAS patients, regardless the intensity of the mucosal disease, do not develop any other systemic symptoms. In recent years, many studies evaluating morphological, histopathological, and immunohistochemical features of RAS and BD have been published [8, 25]. Oh et al. compared the clinical features of RAS and BD and found minor differences [25]. According to this study, BD patients tend to manifest more major aphthae on their oral mucosa, and involvement of more than two sites was more common in BD. Also, exacerbation of oral aphthae during the premenstrual period was more frequent in BD patients. But these results are not definitive for differential diagnosis, and researchers stated that RAS patients should be followed up for potential manifestation of BD [25].

Due to AOU tendency to spontaneous healing and well-known morphology, biopsies are rarely performed, and, due to similar histopathological features of all variants of aphtous oral ulcers, histopathological examination has a limited value in the differential diagnosis. Lymphocytes, macrophages, and neutrophils are observed at the base of an AOU [29]. The infiltrate is more pronounced around the vessels. Although classified as vasculitis, some studies report that most mucocutaneous lesions in BD do not present typical characteristics of an actual vasculitis [7]. Fibrinoid necrosis in the vessel walls is reported to be very rare [5]. At the periphery of the ulcer base, the infiltrate may penetrate into the epidermis. Some recently published direct immunofluorescence (DIF) studies report IgM and C_3_ deposits in perivascular region with or without granular C_3_ deposits at the dermoepidermal junction in the perilesional skin of AOU in Behçet's disease patients [8, 25]. Also in another study, Wilhelmsen et al. evaluated perilesional skin of 23 RAS patients with direct immunoflourescence and found out the immunocomplexes to be absent [30]. Clinical significance of this study requires to be validated by other studies. If validated, this finding may be of utmost value in the differential diagnosis of RAS and BD (see Table 2).

## 3. Genital Ulcers

Genital ulcers manifest in the majority of BD patients [31, 32]. Usually larger than oral aphthae, genital ulcers of BD have similar clinical features. Most common places for genital ulceration are the scrotum and the shaft of the penis in men and the major and minor labia in women. Genital ulcers have irregular borders, are deeper than oral aphthae, and heal with scarring and occasionally causing fistulae extending to the urethra or bladder. Borders of genital ulcers are surrounded by an erythematous rim and fibrinous material, and whitish-yellow pseudomembranes can be found on the ulcer base. Observation of genital ulcers or remaining scars is of diagnostic value. 

Many sexually transmitted diseases (STDs) manifest with genital ulcers, but usually, most STD diagnoses can easily be established with physical examination findings (i.e., lymphadenopathies—bubo formation in chancroid, lack of pain in syphilitic ulcer fever, and malaise). Also, direct observation of pathogenic microorganisms obtained via swaps by Wright, Giemsa stains and cultures for the suspected pathogens from ulcer bases usually make the histopathological evaluation inessential. Histopathology of genital ulcers is similar to that of the oral aphthae. The same mixed infiltrate consisting of neutrophiles, lymphocytes, and macrophages is ever-present at the ulcer base. In conclusion, diagnosis of Behçet's disease genital ulcers is a diagnosis of exclusion (see Table 2).

## 4. Extragenital Ulcers

In some BD patients, cutaneous ulcerations similar to aphtous lesions are reported on different locations other than oral mucosa and external genitalia. Legs, neck [33], and interdigital areas are some of the reported sites. Extragenital ulcers are seen in about 3% of BD patients and observed usually in children [34, 35]. A typical extragenital ulcer is a small, circumscribed, shallow ulcer with a red rim and yellow or grey base. These ulcers may persist for weeks and can be very painful. Due to their infrequency, there are few case reports and fewer studies evaluating these ulcers. Azizlerli et al. reported vasculitis in four cases [33] (see Table 2).

## 5. Erythema-Nodosum-Like Lesions

Nodular lesions located on the lower extremities resembling erythema nodosum are frequently seen in Behçet's disease patients. Erythema-nodosum-like lesions (ENLs) are rather common [36]. ENLs manifest mostly in females. Other than lower extremities, ENLs are reported on face and neck [31]. ENLs do not ulcerate and heal in 2-3 weeks. The main difference between erythema nodosum and ENL is the existence of vasculitis and necrobiosis in the latter. Nodular vasculitis is another condition, which may resemble ENL and can be distinguished by the presence of granulomas and lymphocytic infiltration of subcutis.

There are conflicting results concerning the nature of the vasculitis. Two research groups, Chun et al. [37] and Kim and LeBoit [38], reported “lymphocytic vasculitis” as the dominant pattern. On the other hand, observation of “neutrophilic vasculitis” patterns was also reported [4, 39]. Pathogenesis of ENL vasculitis is unknown. Kaneko et al. [40] reported IgM deposits in the vessel walls in the lesional skin. Some authors believe this vasculitis to be a secondary event to lymphocytic infiltration [37]; others [41] proposed ENL vasculitis as a primary vasculitis (see Table 2).

## 6. Pathergy Reaction

The term “pathergy” is used to define the cutaneous hyperreactivity to minimal trauma. A positive pathergy reaction in BD is characterized by an erythematous, indurated papule at the site of trauma, which usually evolves into a sterile pustule. Pathergy is one of the diagnostic criteria for BD and accepted as a sign for the active disease. Neutrophilic dermatoses (pyoderma gangrenosum, Sweet syndrome, and erythema elevatum diutinum) are also known for positive pathergy reaction. In this conditions, especially in pyoderma gangrenosum (PG), positive pathergy may have different manifestations. Pathergy in PG may describe development of the new skin lesions, the exacerbation of the existing ones following a minor skin trauma, or rapid enlargement of PG after debridement [42, 43]. Pathergy positivity is also reported in chronic myeloid leukemia (CML) patients [42].

The underlying mechanism of pathergy is unknown, and test positivity differs between different countries. Pathergy positivity is observed more frequently in the Mediterranean peninsula and Japan [31]. 

Results of the histopathological studies of pathergy in BD are conflicting. The only consensus on this subject is the presence of an infiltrate consisting of mononuclear cells around dermal vessels at the pathergy site. Some studies revealed the neutrophils as the major constituent of the infiltrate [44], while research groups found percentage of neutrophils relatively low [45]. There are also conflicting results in regard to vasculitis in pathergy of BD. Jorizzo et al. reported leukocytoclastic vasculitis [46]. Ergun et al. studied the change in the histopathological features of pathergy in BD patients and failed to observe a vasculitic pattern [47]. 

Presence of mast cells at the pathergy site is another histopathological feature worth of mentioning [42, 48]. Degranulation of mast cells is suggested to play a role in the pathergy [42, 49].

 In 2009, Kose published a paper evaluating IgG, IgM, IgA, and C_3_ deposits in 108 BD patients by direct immunoflourescence, 44 of the skin samples were obtained from positive pathergy sites, and high deposition rates of IgM, IgA, and C_3_ were found [8], indicating a probable underlying autoimmune mechanism (see Table 2).

## 7. Papulopustular Lesions (PPLs)

Papulopustular lesions (PPLs) are the most common cutaneous manifestation of BD [31, 32]. PPLs are observed on the trunk, face, and extremities. ISG criteria concerning PPLs define them as “pseudofolliculitis or papulopustular lesions; or acneiform nodules observed by physician in postadolescent patients not receiving corticosteroid treatment” and do not clarify the exact nature of lesions. ISG definition of PPL refers to papular lesions on an erythematous base and progressing to sterile pustules [50], but papulopustular lesions of acne are also consistent with this definition, rendering this criteria impractical in the BD diagnosis during the adolescence period. 

To determine whether a papulopustular eruption is a part of BD complex is probably the most challenging part of the diagnostic process. Some authors propose that nonfollicular lesions located in other than face are more specific for Behçet's disease [31] and some propose exclusion of PPL from the diagnostic criteria due to its vague definition [50]. Despite the increasing number of studies evaluating the histopathological features of PPL, the exact nature of PPL is still needed to be established.

The results of these histopathological studies are also contradictory. Certain study results indicate the presence of vasculitis [5, 31, 32, 50] in the histopathological sections and suggest that the term “pseudofolliculitis” is a misnomer and that it should be dropped [5, 31], while some authors report perifolliculitis or suppurative folliculitis observed during the histopathological evaluations and consider histopathological sections of little help in the differential diagnosis [51]. More recent studies support the role of vasculitis in the evolution of Behçet's disease papulopustular lesions [5, 10, 31]. Intraepidermal pustules, spongiosis, neutrophil or lymphocyte exocytosis, basal keratinocyte vacuolization, edema in dermis, lymphohistiocytic or neutrophilic inflammatory infiltration between collagen fibers and perivascular areas, fibrin deposition within vessel walls, endothelial swelling, and erythrocyte extravasation are reported as the histopathological features of Behçet's disease PPL [10]. To further clarify the presence of vasculitis, PPLs were evaluated by immunofluorescence [8, 9, 11]. A study by İlknur et al. failed to find any difference between the direct immunofluorescence findings of eighteen Behçet's disease patients and sixteen patients with bacterial folliculitis and five patients with acne [9]. In other two studies [8, 11] (seventeen and one hundred eight patients, resp.) immunoreactant deposition in the lesional and nonlesional skin of the BD patients was evaluated and significant deposition, especially IgM in the lesional skin was reported, supporting the immune-mediated vasculitis hypothesis (see Table 2).

## 8. Thrombophlebitis

Behçet's disease may also affect major vessels. Although there are conflicting views about the presence of an actual vasculitis in the mucocutaneous lesions of BD, actual vasculitis of major vessels is welldocumented [7, 31]. Involvement of any vessel is possible, but venous system seems to be the primary target [52, 53] and subcutaneous thrombophlebitis is reported to be the most frequent in the venous involvement [31]. Exact pathogenesis is unknown, but Th-1 type inflammatory response is suspected just as in other vasculitides like Wegener Granulomatosis and temporal arteritis. Unlike other primary vasculitides, major vascular involvement of BD predominantly affects males [7]. 

Subcutaneous thrombophlebitis is another common cutaneous manifestation of BD. Erythematous, tender nodules occur on the site of venous involvement. Consistent with the size of the affected vessel, an erythematous, lineer hardening can also be palpated. During an activation period, several separate nodules may manifest consecutively on different localizations since multiple vascular segments may be involved, so BD must always be included in the differential diagnosis of “superficial migratory thrombophlebitis.” Superficial thrombophlebitis may also herald the coexisting major vessel vasculitis and thrombotic condition [54, 55]. Histopathology of superficial thrombophlebitis is nonspecific. A thrombi in the vascular lumen and concomitant perivascular infiltrate consisting of mononuclear cells are the classical histopathological features (see Table 2).

## 9. Rare Cutaneous Lesions

There is an increasing number of reports about other coexistent cutaneous lesions in Behçet's disease patients. Among these are; erythema-multiforme-like lesions [56], polyarteritis nodosa-like lesions [57], pernio-like lesions [58], Sweet syndrome [59], necrotizing folliculitis [60], and necrotizing cutaneous small vessel vasculitis. Since reports of similar cases are so rare, association between BD and these skin lesions is not clear and they can be coincidental [31]. No extraordinary histopathological findings in these cases were reported. 

Abnormalities observed in nailfold capillaroscopy are an intriguing and recently defined aspect of BD. Unlike the aforementioned cutaneous lesions, abnormalities in the periungual vessels were observed in relatively large patient populations. Movasat et al. described enlarged capillaries (26%), hemorrhages (16%) in the nail folds of 128 patients with BD [61], and suggested high blood pressure due to Behçet's disease major vessel involvement as the probable underlying factor.

## 10. Conclusion

Diagnosis of BD still depends of the clinican's ability to recognize various, nonspecific mucocutaneous lesions and this nonspecific character of lesions may be a major problem during the diagnostic process (e.g., whether the papulopustular lesions of a patient with recurrent AOU belong to diagnostic criteria). In recent years, histopathological and immunohistochemical evaluation of BD mucocutaneous lesions of B has become focus of many research groups, aiming to enhance the diagnostic value of these lesions. Although there are some contradictory reports, there are increasing reports supporting an underlying immune-mediated vasculitis in the BD mucocutaneous lesions. Leukocytoclastic vasculitis, fibrinoid necrosis of postcapillary venules, or perivascular neutrophilic accumulations are some of the reported patterns in the early stages of the cutaneous lesions [31]. In conclusion, for the differential diagnosis of mucocutaneous lesions of BD (e.g., recurrent oral aphthae, papulopustular eruptions), immunoflourescence methods seem promising.

## Figures and Tables

**Table 1:** Diagnostic criteria of Behçet's disease defined by International Study Group.

Mucocutaneous lesions	Description
Recurrent oral ulceration	Minor, aphthous, major aphthous, or herpetiform ulceration observed by physician or patient that recurred at least 3 times in one 12-month period

Plus 2 of the following criteria:	
Recurrent genital ulceration	Aphthous ulceration or scarring observed by physician or patient
Eye lesions	Anterior uveitis, posterior uveitis, cells in vitreous on slit lamp examination, or retinal vasculitis observed by ophthalmologist
Cutaneous lesions	Erythema nodosum, pseudofolliculitis, papulopustular lesions, or acneiform nodules observed by physician in postadolescent patients not receiving corticosteroid treatment
Positive pathergy test	Read by physician at 24 to 48 hours

**Table 2:** An overview of the reported histopathological and immunoflourescence features of Behçet's disease common mucocutaneous lesions.

Mucocutaneous lesions	Reported histopathological features
Recurrent oral Aphthae	Lymphocytes, macrophages, neutrophils at the base of the ulcer, sometimes penetrating epidermis at the periphery
	Similar infiltrate at the perivascular regions in dermis fibrinoid necrosis of vessel walls (rare)
	Also granular IgM and C_3_ deposits in dermoepidermal junction and in perivascular regions (in RAS, no deposits of immunoreactants) [30]

Genital ulceration	Similar histopathological features to oral aphthae

Erythema-nodosum-like lesions	Neutrophilic vasculitis
	Lymphocytic vasculitis
	Necrobiosis
	IgM deposits at the vessel walls [40]

Pathergy reaction	Perivascular infiltrate of mononuclear cells
	Vasculitis (neutrophilic, leukocytokclastic) (+/−)
	Presence of mast Cells.
	IgM, IgA, and C_3_ deposits

Papulopustular lesions	Intraepidermal pustules, spongiosis, neutrophil/lymphocyte exocytosis, and basal keratinocyte vacuolization,
	Edema in dermis, lymphohistiocytic/neutrophilic inflammatory infiltration between collagen fibers, and perivascular areas
	Vasculitis (+/−)

Thrombophlebitis	Thrombi in the vessel lumen
	Perivascular infiltrate of mononuclear cells
